# Ethylene negatively regulates aluminium-induced malate efflux from wheat roots and tobacco cells transformed with *TaALMT1*

**DOI:** 10.1093/jxb/eru123

**Published:** 2014-03-25

**Authors:** Qiuying Tian, Xinxin Zhang, Sunita Ramesh, Matthew Gilliham, Stephen D. Tyerman, Wen-Hao Zhang

**Affiliations:** ^1^State Key Laboratory of Vegetation and Environmental Change, Institute of Botany, Chinese Academy of Sciences, Beijing 100093, China; ^2^ARC Centre of Excellence in Plant Energy Biology, School of Agriculture, Food and Wine, Waite Research Institute, University of Adelaide, Glen Osmond, SA 5064, Australia; ^3^Research Network of Global Change Biology, Beijing Institutes of Life Science, The Chinese Academy of Sciences, Beijing, P. R. China

**Keywords:** Ethylene, aluminium tolerance, malate efflux, ALMT1, wheat (*Triticum aestivum* L.).

## Abstract

An important mechanism for Al^3+^ tolerance in wheat is exudation of malate anions from the root apex through activation of malate-permeable TaALMT1 channels. Here, the effect of ethylene on Al^3+^-activated efflux of malate was investigated using Al^3+^-tolerant wheat genotype ET8, which has high expression of *TaALMT1*. Exposure of ET8 plants to Al^3+^ enhanced ethylene evolution in root apices. Treatment with the ethylene synthesis precursor 1-aminocyclopropane-1-carboxylic acid (ACC) and ethylene gas suppressed Al^3+^-induced malate efflux from root apices, whereas the intracellular malate concentrations in roots were not affected. Malate efflux from root apices was enhanced in the presence of Al^3+^ by two antagonists of ethylene biosynthesis, aminoethoxyvinylglycine (AVG) and 2-aminoisobutyric acid (AIB). An increase in Al accumulation in root apices was observed when treated with ACC, whereas AVG and AIB suppressed Al accumulation in root apices. Al^3+^-induced inhibition of root elongation was ameliorated by pretreatment with AIB. In addition, ethylene donor (Ethrel) also inhibited Al^3+^-induced malate efflux from tobacco cells transformed with *TaALMT1*. ACC and the anion-channel blocker niflumate had a similar and non-additive effect on Al-induced malate efflux from root apices. Treatment of ET8 plants with ACC enhanced expression of *TaALMT1*, suggesting that the inhibitory effect of ethylene on Al-induced malate efflux is unlikely to occur at the transcriptional level. These findings indicate that ethylene may behave as a negative regulator of Al^3+^-induced malate efflux by targeting TaALMT1-mediated malate efflux by an unknown mechanism.

## Summary

Exudation of malate is an important mechanism underlying tolerance of wheat to aluminium toxicity. Here we show that ethylene is involved in regulation of ALMT1-dependent malate efflux from wheat roots.

## Introduction

Aluminium (Al) is the most abundant metal in the Earth’s crust. Fortunately, the majority of Al occurs in the non-toxic form of aluminosilicate. However, Al is hydrolysed into phytotoxic Al^3+^ cations in acidic environments, and becomes a major constraint for crop growth and yield in acid soils (Kochian, 1995). Inhibition of root elongation is one of the earliest and most distinct symptoms of Al^3+-^toxicity that can be easily observed in solution culture (Zhang and Rengel, 1999). Although Al^3+^ can induce a rapid change in cell division in maize (Doncheva *et al.*, 2005), the rapid suppression of root elongation by Al^3+^ within 1h of exposure to Al^3+^ (Zhang and Rengel, 1999) suggests that Al^3+^-induced inhibition of root growth probably results from arrest of cell elongation (Horst, 1995; Matsumoto, 2000). It has been established that the root apex, particularly the distal transition zone, is a critical site for perception of Al^3+^ and in determining whether a plant exhibits tolerance to Al^3+^ (Ryan et al, 1993; Sivaguru and Horst, 1998). Extensive studies have demonstrated that numerous molecular and physiological processes are targeted by Al^3+^, such as Ca^2+^-dependent signalling cascades, cytoskeleton dynamics (see reviews of Matsumato, 2000; Rengel and Zhang, 2003), phytohormones (auxin, Kollmeier *et al.*, 2000; Illes *et al.*, 2006; Shen *et al.*, 2008; ethylene, Sun *et al.* 2010), and nitric oxide (Wang and Yang 2005; Illes *et al.*, 2006; Tian *et al.*, 2007). However, the primary mechanisms underlying Al^3+^ toxicity in plants remain largely controversial and elusive.

In contrast to Al^3+^ toxicity, substantial progress has been made in our understanding of Al^3+^ tolerance (see reviews of Ryan *et al.*, 2011; Delhaize *et al.*, 2012). An important tolerance mechanism is the exudation of carboxylic anions (malate, citrate, oxalate) that can complex extracellular Al^3+^ (Ryan *et al.*, 2001; Ryan *et al.*, 2011; Ma *et al.* 2001). In wheat, malate is exuded from the root apex upon exposure to Al^3+^ (Delhaize *et al.*, 1993*b*; Ryan *et al.*, 1995). Further studies have revealed that Al^3+^-induced exudation of malate is mediated by anion channels permeable to malate (Ryan *et al.*, 1997; Zhang *et al.*, 2001, 2008). Sasaki *et al.* (2004) identified that a membrane protein ALMT1 underpins the Al^3+^-induced malate exudation from root apices in wheat. Heterologous expression of the *TaALMT1* gene in *Xenopus oocytes* and in tobacco BY2 cells revealed the kinetic properties of malate transport (Sasaki *et al.*, 2004; Zhang *et al.*, 2008 Piñeros *et al.*, 2008). When expressed in barley (Delhaize *et al.*, 2004) and tobacco BY2 cells (Sasaki *et al.*, 2004; Zhang *et al.*, 2008), TaALMT1 conferred an Al^3+^-activated efflux of malate that improved resistance to Al^3+^. Transporters homologous to ALMT1 have been identified to mediate Al^3+^-induced malate efflux in species such as *Arabidopsis thaliana* (Hoekenga *et al.*, 2006), rye (Collins *et al.*, 2008), barley (Gruber *et al.*, 2011), *Brassica napus* (Ligaba *et al.*, 2006), and soybean (Liang *et al.*, 2013). The function and regulation of TaALMT1 have been characterized at both transcriptional and post-transcriptional levels. For instance, the promoter characteristics (Sasaki *et al.*, 2006; Ryan *et al.*, 2010), membrane topology (Motoda *et al.*, 2007), N-terminal and C-terminal domains (Ligaba *et al.*, 2013; Furuichi *et al.*, 2010), and putative protein phosphorylation sites have been shown to be involved in regulating the function of TaALMT1 (Osawa and Matsumoto, 2001; Ligaba *et al.*, 2009). Although numerous studies have investigated the mechanisms of Al^3+^-induced malate efflux mediated by ALMT1, it is unclear how Al^3+^ activates the ALMT1 channels (Furuichi *et al.*, 2010; Ryan *et al.*, 2011; Ligaba *et al.*, 2009, 2013).

Our previous studies revealed that Al^3+^ evokes ethylene evolution from root apices of *Lotus japonicas* (Sun *et al.*, 2007) and *Arabidopsis* (Sun *et al.*, 2010). In vascular plants, ethylene is produced from methionine through *S*-adenosyl-l-methionine and 1-aminocyclopropane-1-carboxylic acid (ACC), catalysed by ACC synthase (ACS) and ACC oxidase (ACO), respectively (Kende, 1993). We demonstrated that the Al^3+^-induced suppression of root elongation is negatively correlated with Al^3+^-elicited ethylene production, such that inhibition of ethylene biosynthesis with antagonists markedly alleviates the inhibitory effect of Al^3+^ on root growth (Sun *et al.*, 2007). In *Arabidopsis*, we further demonstrated that the Al^3+^-induced ethylene may act as a signal to alter auxin distribution by targeting PIN2 and AUX1, leading to suppression of root growth (Sun *et al.*, 2010). There is emerging evidence indicating that ethylene is involved in regulation of several membrane transporters at the transcriptional level, including both high- and low-affinity nitrate transporters in *Arabidopsis* (Tian *et al.*, 2009) and oilseed rape (Leblanc *et al.*, 2008), a high-affinity potassium transporter in *Arabidopsis* (Jung *et al.*, 2009), high-affinity phosphate transporters in *Arabidopsis* (Lei *et al.*, 2011) and *Medicago falcatula* (Li *et al.*, 2011), and an iron transporter in *Arabidopsis* (Garcia *et al.* 2010). In addition to regulation of nutrient transporters at the transcriptional level, ethylene can activate Ca^2+^-permeable cation channels, leading to an increase in cytosolic Ca^2+^ activity in tobacco BY2 cells (Zhao *et al.*, 2007). Given that Al^3+^ triggers ethylene production in roots of some plants and ethylene can regulate some ion channels, we explored the possibility that ethylene may be involved in regulation of Al tolerance by targeting ALMT1-mediated malate efflux. Our results showed that Al^3+^-induced malate efflux from root apices and *TaALMT1*-expressing tobacco BY2 cells was correlated with ethylene production, suggesting the regulatory role of ethylene in TaALMT1-dependent tolerance to Al^3+^.

## Materials and methods

### Plant materials and growth conditions

Seeds of ET8, the Al-tolerant genotype of wheat *Triticum aestivum* L. (Delhaize *et al.*, 1993*b*), were surface-sterilized by incubation for 1min in 75% ethanol, rinsed with sterile distilled water followed by exposure to 10% (v/v) sodium hypochlorite for 20min, and then washed with sterile water. The seeds were transferred to 100ml flasks (10 seeds/flask) containing 40ml sterile 0.2mM CaCl_2_, pH 4.5 (control solution). Seed germination occurred during incubation at 22–28 ºC for 4–5 d on an orbital shaker set at 100rpm.

### Determination of ethylene production

Roots of five-day-old seedlings were exposed to solutions containing 0, 50, and 200 μM AlCl_3_ (pH 4.5) with basal composition of 0.2mM CaCl_2_ for 2h, before root apices (about 2cm long) of about 0.3g were excised. To minimize the wounding effect, the excised roots were placed into 5ml gas-tight vials containing 0.5ml of agar medium (0.7% agar) for 1h, and then the vials were sealed with a gas-tight stopper. The excised roots were kept moist during the 1-h period. One millilitre of headspace gas was taken from the vials after 1h collection time and injected into a gas chromatograph (GC) equipped with an alumina column (GDX502) and a flame ionization detector (GC-7AG; Shimadzu Japan) for determination of the ethylene concentration.

### Staining Al by haematoxylin and determination of Al in root apices

Al distribution in root apices was visualized using Lumogallion following protocols described by Delhaize *et al.* (1993*a*). Briefly, root apices were first exposed to 0 μM and 10 μM 1-aminocyclopropane-1-carboxylic acid (ACC) for varying durations, and then incubated in 20 μM AlCl_3_ (pH 4.5) for 30min. After rinsing thoroughly with deionized water, they were transferred to 100ml solutions containing 0.2g haematoxylin and 2mg KIO for 30min. The roots were photographed after being washed with deionized water.

To examine the effect of ethylene on Al accumulation, five-day-old wheat seedlings were first exposed to either 10 μM aminoethoxyvinylglycine (AVG) or 50 μM 2-aminoisobutyric acid (AIB) for 6h and then incubated in 20 μM AlCl_3_ for 30min. Control roots that were not treated with AVG and AIB were also exposed to an identical Al solution. Al contents in root apices were determined following the protocols used by Rangel *et al.* (2007). Briefly, about 20 root apices that were thoroughly rinsed with 0.2mM CaCl_2_ (pH 4.5) were transferred into 2ml Eppendorf reaction vials and digested in 500 μl ultra-pure HNO_3_ (65%) on a rotary shaker for 24h. The digestion was completed by heating the samples in a water bath at 80 ºC for 20min. Samples were diluted by addition of 1.5ml distilled water after cooling. All samples were passed through a 0.45 μM filter (Millipore, USA). Al concentration in the extract solution was measured by Inductively Coupled Plasma Emission Spectrometer (ICP-OES, Thermo Electron Corporation, USA)

### Determination of malate efflux and intracellular malate contents

Malate exudation from root apices was determined according to the method of Ryan *et al.* (1995) with minor modifications. Root apices (1cm) were excised with a razor blade from plants incubated in control solution (0.2mM CaCl_2_, pH 4.5). Thirty root-apices for each measurement were transferred into 5ml vials and washed three times with control solution to remove malate released from the cut surface. Excised root apices were exposed to control solution and to solutions supplemented with 10 μM ACC for 2h, and then incubated in l ml solution containing 20 μM AlCl_3_ for another 2h. During the treatment, the vials were placed on a reciprocal shaker (100rpm). After 2h, the solution was collected for malate analysis. To determine the effect of ethylene gas on malate efflux, the excised root apices were transferred into 5ml vials containing 0.15ml control solution. The solubility of ethylene in solution is very low, thus a minimum volume of solution was used to maximize effective ethylene concentration. The vials were sealed and 1ml ethylene gas (500 ppm) or air was injected into the vials. After treatment for 2h, root apices were exposed to 0 or 200 AlCl_3_ for another 2h. To study the effect of ethylene synthesis inhibitors AVG and AIB on malate efflux, roots were incubated in solutions containing 10 μM AVG and 50 μM AIB for 6h. Root apices were excised and placed into vials containing 1ml 200 μM AlCl_3_ to collect malate for 1h. To determine the additive effect of ACC and the anion channel blocker niflumic acid (NA), thirty root apices were treated with 1ml solutions containing various concentrations of NA (0, 2, 5, 10, 20 μM) or ACC (0, 5, 10, 15, 20, 30, 50 μM) and 200 μM AlCl_3_ for 1h.

Malate concentrations of the exudation solution were determined following protocols used by Delhaize *et al.* (1993*b*). One ml sample solution was incubated with 1ml buffer (0.5M Gly, 0.4M hydrazine, pH 9.0) and 0.1ml NAD. After 5min, the reaction solutions were used to determine the absorption at 340nm (the first A_340_). The reaction mixture was then incubated for 40min after the addition of 5 µL malate dehydrogenase (MDH). The production of NADH leads to the increase in A_340_. The change of A_340_ before and after addition of MDH was used to calculate malate content.

Malate efflux from tobacco BY2 cells was measured as described by Zhang *et al.* (2008). BY2 cells (*Nicotiana tabacum* L. cv. Samsun, a cell line SL) transformed with the *TaALMT1* gene from wheat, or an empty vector (Sasaki *et al.*, 2004) were grown in MS media. The transgenic BY2 cells were grown in MS media solution on a rotary shaker until the logarithmic phase of growth. Aliquots of suspension containing approximately 1g of cells were centrifuged and the cells were gently resuspended in 15ml of 3mM CaCl_2_ and 3mM sucrose (pH 4.5). Aliquots were then collected and cells resuspended in the above solution treated with or without added treatments at approximately 0.15g FW per 10ml. Treatments included 10 μM Ethrel, 100 μM AlCl_3_ (pH 4.5) or 10 μM Ethrel plus 100 μM AlCl_3_ (pH 4.5) for 60min. After the treatment, the suspensions were centrifuged and malate concentrations in the supernatant were assayed as described above.

To measure malate concentrations in root apices, thirty root apices were homogenized in liquid N_2_ and extracted using a pestle in 1ml of ice-cold 0.6 N perchloric acid after washing thoroughly with control solutions. The extract was centrifuged at 15000 ×g for 5min and 0.9ml of supernatant solution was collected and neutralized with 80 μL of K_2_CO_3_ (69g 100ml^–1^). The solution was centrifuged at 15000 ×g for 5min. The contents of malate were assayed as described above after mixing 0.5ml of the supernatant with 0.5ml distilled water.

### Measurements of root elongation

Roots of 5-day-old seedlings were exposed to solutions containing different concentrations of AlCl_3_ (0, 10, 20, 50, 100 μM, pH 4.5) or ACC (0, 0.01, 0.1, 1, 10 μM, pH 4.5) for 24h. Root elongation was determined by a ruler (± 0.5mm) before and after treatments. To study the short-term effect of AlCl_3_ and ACC on root elongation, ET8 seedlings were incubated in control solution and solutions containing 10 μM ACC or 20 μM AlCl_3_ (pH 4.5) for 1h, and root length was measured under a microscope (SZX12, OLYMPUS, Japan) before and after treatments. To measure the effect of ethylene synthesis inhibitors (AVG, AIB) on root elongation, roots of 5-day-old seedlings were pretreated with control solution or solutions containing 10 μM AVG or 50 μM AIB for 6h, and then exposed to 0 μM or 20 μM AlCl_3_ (pH 4.5) for another 2h. Root length was measured under microscope before and after exposure to AlCl_3_.

### Analysis of TaALMT1 gene expression

Real-time PCR was used to study the expression patterns of *TaALMT1* in ET8 in response to AlCl_3_ and ACC. After exposure of ET8 to AlCl_3_ (20 μM) or ACC (10 μM) for varying time periods (0, 2, 6, 24h), approximately 50 root apices were excised and frozen in liquid N_2_. RNA was isolated using RNAiso Plus reagent (TaKaRa). The RNA was reverse-transcribed into first-strand cDNA with PrimeScript RT reagent Kit (TaKaRa): 0.5 μg of total RNA, 1 μL of 5×DNA Eraser Buffer, 0.5 μL of gDNA Eraser, and DEPC-H_2_O to 5 μL. The solution was incubated at 42 ºC for 2min to remove any contaminating genomic DNA. Each reaction was adjusted to 10 μL by adding 2.0 μL of 5×Primer Scrip buffer, 0.5 μl Primer Script RT Enzyme Mix I, 0.5 μl RT-Primer Mix, and DEPC-H_2_O. The reverse-transcription was performed for 30min at 37 ºC and terminated at 85 ºC for 5 s. Three endogenous genes *TaActin*, *TaTubulin*, and *TaGAPDH* were used as control genes. *TaALMT1* expression relative to the control genes was determined by real-time quantitative RT-PCR (qRT-PCR) on an ABI StepOne Plus instrument. Each reaction contained 5.0 μl 2×UltarSYBR Mixture (With ROX) reagent (Cwbio), 1.5 μl cDNA samples, and 1.2 μl of 10mM gene-speciﬁc primers in a ﬁnal volume of 10 μl. Thermocycling conditions were 95 ºC for 10min followed by 40 cycles of 95 ºC for 30 s, 55 ºC for 30 s, and 72 ºC for 30 s. The primers used for real-time PCR of *TaALMT1* were those used by Sasaki *et al.* (2006): 5′-AAGAGCGTCCTTAATTCG-3′ and 5′-CCTTACATGATAGCTCAGGG-3′. Three endogenous genes of *TaActin*, *TaTubulin*, and *TaGAPDH* were amplified with the following primers: TaActin-F (5′-CTATCCTTCGTTTGGACCTT-3′), TaActin-R (5′-AGCGAGCTTCTCCTTTATGT-3′), TaTubulin-F (5′-TCCATGTCGTCGACTGGTGC-3′), TaTubulin-R (5′-TCC TCGTAGTCCTTCCTCCCAG-3′), TaGADPH-F (5′-GTTGA GGGTTTGATGACCAC-3′), TaGADPH-R (5′-TCGGACTCC TCCTTGATAGC-3′). Sequencing of PCR products was used to confirm whether the primers amplified the target genes. Three biological and three technological repeats were performed in RT-PCR. The relative expression level was analysed by the comparative C_T_ method.

## Results

### Al-stimulated ethylene evolution from root apices

**Fig. 1.**
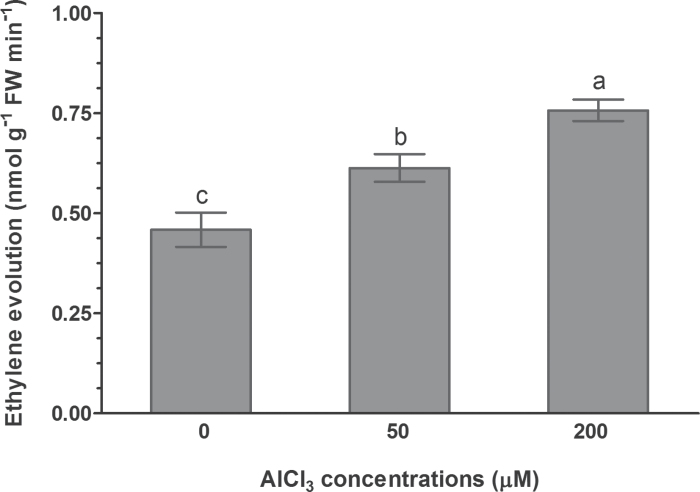
The effect of Al on ethylene evolution in root apices of Al-tolerant ET8 wheat plants. ET8 seedlings were exposed to 0, 50, and 200 μM AlCl_3_ (pH 4.5) for 2h, and the ethylene concentrations were measured by gas chromatography. The control solution contained 0.2mM CaCl_2_ (pH 4.5). Data are the mean ± SE of four replicates. Data with different letters indicate significant different (*P*<0.05) between treatments.

To establish a link between ethylene and Al^3+^-induced malate efflux from wheat root apices, the effect of Al^3+^on ethylene evolution from excised root apices of ET8 plants was determined. Similar to *L. japonicas* (Sun *et al.*, 2007) and *A. thaliana* (Sun *et al.*, 2010), exposure of 5-day-old ET8 seedlings to solutions containing 50 and 200 μM AlCl_3_ for 2h (pH 4.5) led to an increase in ethylene evolution above control levels (Fig. 1). The magnitude of ethylene evolution from root apices was positively dependent on Al^3+^ concentrations, such that an increase in ethylene evolution was increased by 33.6% and 65.0% after treatment with 50 and 200 μM AlCl_3_, respectively.

### Ethylene inhibited Al^3+^-induced malate efflux from root apices

**Fig. 2.**
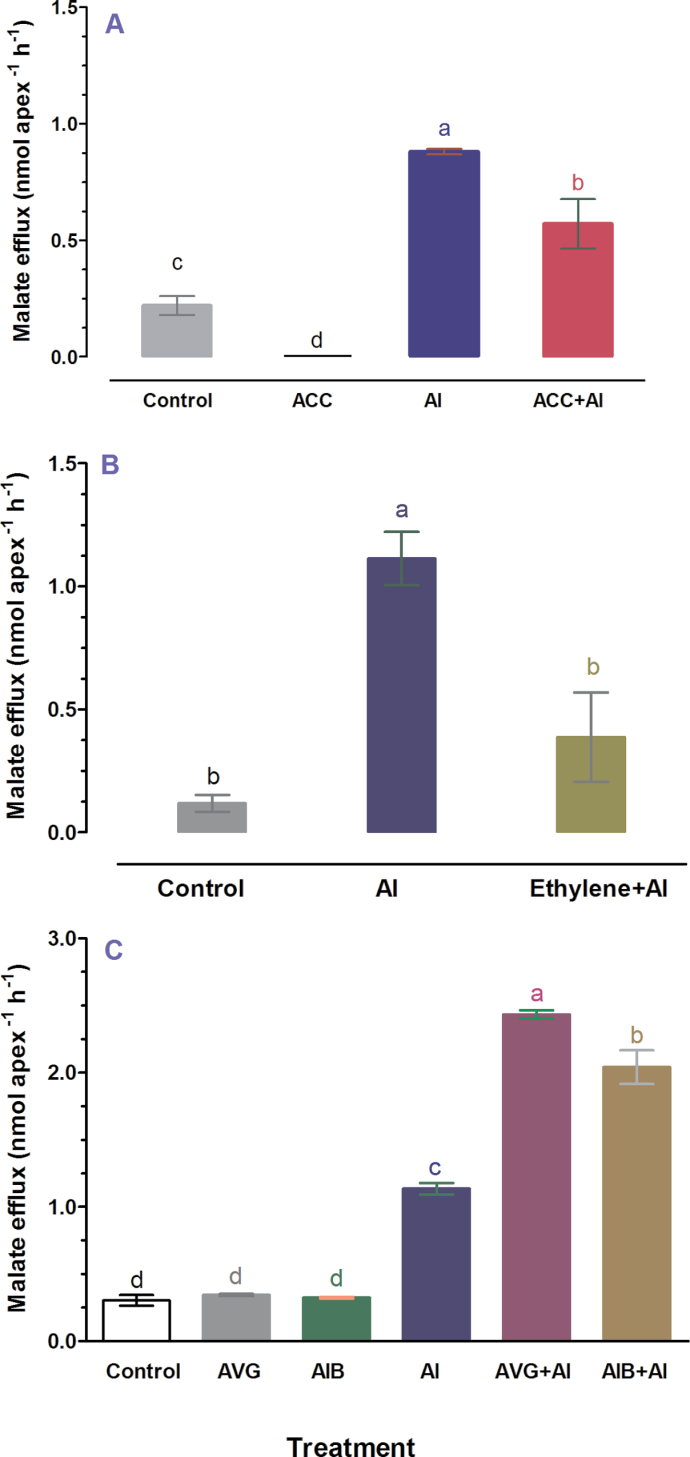
Effect of ethylene biosynthesis precursor (ACC), ethylene gas (C_2_H_4_), ethylene synthesis inhibitors (AVG, AIB) and Al on malate efflux from the root apex. (A) Thirty root apices (1cm in length) of five-day-old seedlings were exposed to 200 μM AlCl_3_ for 2h after first being incubated for 2h in and/or 10 μM ACC. (B) Thirty root apices were transferred to 5ml gas-tight vials containing 100 nl ml^–1^ ethylene gas for 2h and then incubated to 200 μM AlCl_3_ for 2h. (C) Root apices were exposed to 200 μM AlCl_3_ for 1h, after seedlings were incubated in 10 μM AVG and 50 μM AIB for 6h. The malate in solution was measured by enzyme method. Data are the means ± SE of four replicates. The different letters indicate significant difference at *P*<0.05 tested with SAS Software.

Previous studies revealed that Al^3+^ can induce a rapid malate efflux from ET8 root apices (Ryan *et al.*, 1995). The observation that Al^3+^ also increased ethylene production in root apices of ET8 seedlings prompted us to examine whether the Al^3+^-induced ethylene is involved in regulation of malate efflux from root apices. To evaluate the role of ethylene in malate efflux from wheat root apices, we first examined the effect of ethylene biosynthesis precursor ACC on malate efflux. As shown in Fig. 2A, ACC abolished the basal level of malate efflux from root apices. A marked increase in malate efflux from root apices was observed upon exposure to Al^3+^, and the Al^3+^-induced malate efflux was significantly suppressed by ACC (Fig. 2A). To validate that the inhibitory effect of ACC on Al-induced malate efflux is related to ethylene, the effect of ethylene gas on Al^3+^-induced malate efflux from ET8 root apices was further studied by exposing the roots to ethylene gas before treatment of roots with Al^3+^. Similar to ACC treatment, there was a significant reduction in Al^3+^-induced malate efflux from root apices when treated with ethylene gas (Fig. 2B). ACS and ACO are two key enzymes catalysing ethylene production in vascular plants. In contrast to ACC and ethylene gas, AVG and AIB (ACS and ACO inhibitors, respectively), stimulated Al^3+^-induced malate efflux from root apices (Fig. 2C), whereas AVG and AIB had no effect on malate efflux from root apices in the absence of Al^3+^ (Fig. 2C). These results suggest that ethylene may negatively regulate Al^3+^-induced malate efflux from wheat roots.

### Ethylene and niflumic acid had similar effect on Al^3+^-induced malate efflux

**Fig. 3.**
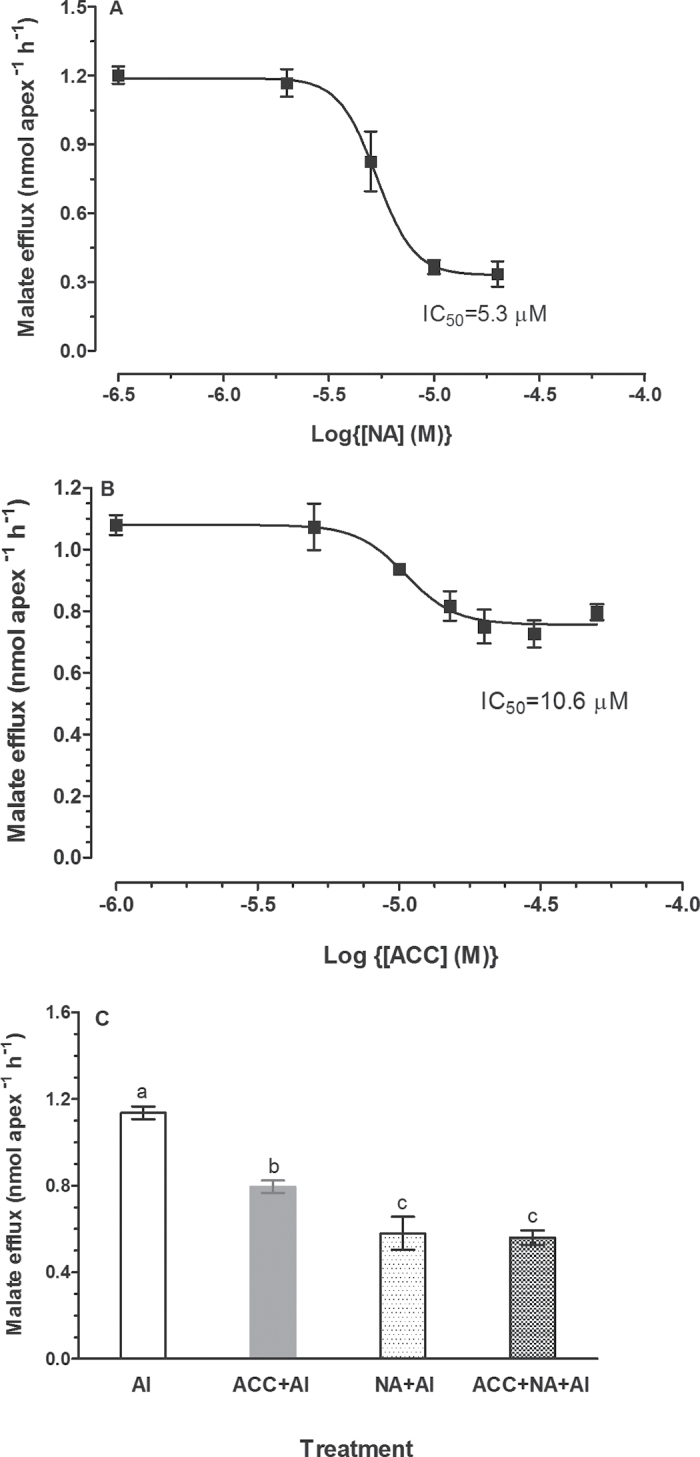
Effect of anion channel blocker (NA), ethylene biosynthesis precursor (ACC), and Al on malate efflux in root apices of ET8 plants. (A) Excised root apices from five-day-old seedlings were washed in control solution and treated for 1h in solutions containing 200 μM AlCl_3_ and various concentrations of NA (0, 2, 5, 10, 20 μM). (B) Root apices were exposed to 200 μM AlCl_3_ and various concentrations of ACC (0, 5, 10, 15, 30 μM) for 1h. (C) Root apices were incubated in solutions containing 11 μM ACC, 9 μM NA and 200 μM AlCl_3_ for 1h. Data are the means ± SE of four replicates. The different letters indicate significant difference at *P*<0.05 tested with SAS Software.

It has been shown that Al^3+^-induced malate efflux is mediated by anion channels (Ryan *et al.*, 1997; Zhang *et al.*, 2001, 2008). The anion channel blocker niflumic acid (NA) inhibits Al^3+^-induced malate efflux from wheat root apices (Ryan *et al.*, 1995) and blocks malate-permeable channels (Zhang *et al.*, 2001, 2008). The effect of NA and ACC on Al^3+^-induced malate efflux from ET8 root apices was compared by analysing their dose-response curves. Our results show that both NA and ACC inhibited Al^3+^-induced malate efflux, and the IC_50_ (concentration of inhibitor producing 50% inhibition) values for NA and ACC were not significantly different (Fig. 3A, B). However the extent of inhibition was larger for NA (72.1%) compared with ACC (31.0%). Moreover, there was no additive effect of ACC and NA on Al^3+^-induced malate efflux, as treatment with NA had an identical effect on malate efflux to treatment with NA and ACC together (Fig. 3C). These results suggest the inhibition of Al^3+^-activated malate efflux by ethylene may result from blockade of malate-permeable anion channels.

### Intracellular malate concentrations in roots were not affected by ethylene

**Fig. 4.**
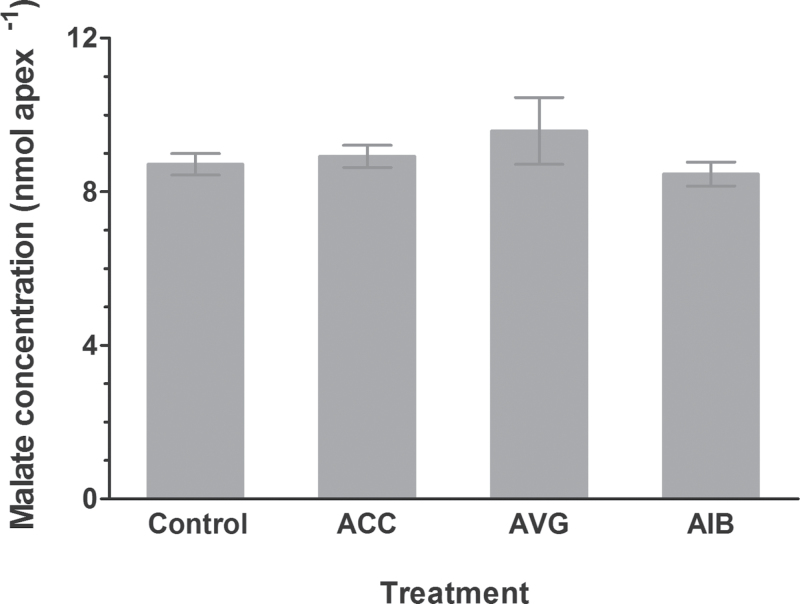
Effect of ACC, AVG, and AIB on malate concentrations of root apices. ET8 seedlings were grown in control solution for 5 d, then transferred to 10 μM ACC (ethylene biosynthesis precursor), 10 μM AVG, and 50 μM AIB (ethylene synthesis inhibitors) for 6h. Root apices (1cm length) were excised and washed by control solution for three times to measure internal malate concentrations. Data are the means ± SE of four replicates. The same letters indicate no significant difference at *P*<0.05 tested with SAS Software.

In addition to malate efflux, we also determined the effect of ACC and ethylene synthesis inhibitors (AVG, AIB) on intracellular malate concentrations of ET8 root apices. Our results showed no effect of ACC, AVG, and AIB on malate concentrations in ET8 root apices (Fig. 4). These results reveal that ethylene negatively regulates Al^3+^-induced malate efflux from ET8 root apices.

### Ethylene inhibited Al^3+^-induced malate efflux from transgenic tobacco suspension cells

**Fig. 5.**
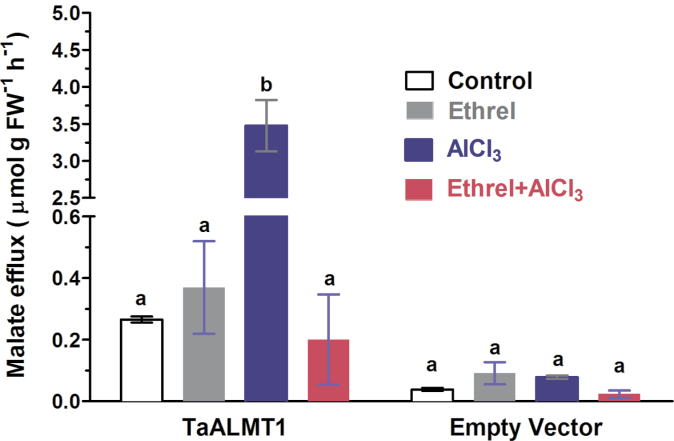
Effect of ethylene donor Ethrel (10 μM) and Al^3+^ (100 μM AlCl_3_) on malate efflux from tobacco BY2 suspension cells transformed with TaALMT1 or empty vector. Control solution consisted of 3mM CaCl_2_, 3mM sucrose, 5mM MES/BTP, pH 4.5. AlCl_3_ was added in the last hour of 3-h incubation, whereas Ethrel was present for either 3h, or for 2h before 1h + AlCl_3._ Different letter indicates significant difference (*P*<0.05).

Previous studies showed that expression of TaALMT1 in tobacco suspension cells resulted in Al^3+^-induced malate efflux (Zhang *et al.*, 2008). To further evaluate the role of ethylene in regulation of Al^3+^-dependent malate efflux, malate efflux from tobacco suspension cells exposed to Al and ethylene was determined. Malate efflux from the transgenic tobacco cells was significantly enhanced by exposure to 100 μM AlCl_3_ (pH 4.5), and the Al^3+^-induced malate efflux was suppressed by 94% when the cells were pretreated with 10 μM Ethrel (Fig. 5). A similar inhibitory effect of Ethrel on Al^3+^-induced malate efflux from the tobacco suspension cells was also found when the suspension cells were treated with Al^3+^ and 10 μM Ethrel simultaneously. The same concentration of Ethrel had no effect on malate efflux from the tobacco cells expressing either empty vector or TaALMT1 (Fig. 5). These results indicate that the inhibition of Al^3+^-induced malate efflux from ET8 root apices by ethylene is likely to result from the suppression of TaALMT1-mediated malate efflux.

### Ethylene enhanced Al accumulation in root apices

**Fig. 6.**
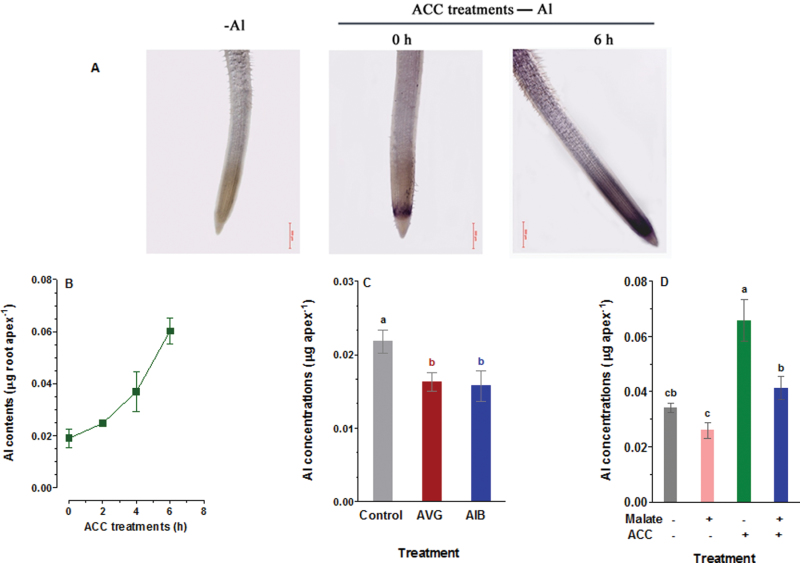
Effect of ethylene biosynthesis precursor (ACC), ethylene synthesis inhibitors (AVG, AIB) and malate on Al accumulation in root apices. (A) ET8 seedings were first exposed to 0 μM and 10 μM ACC for 6h followed by 0 μM and 20 μM AlCl_3_ for 30min, then stained by haematoxylin. (B) Al contents in root apices were measured after being pretreated with 10 μM ACC for varying time periods (0, 2, 4, 6h). (C) The roots of ET8 seedlings were treated for 6h with 10 μM AVG and 50 μM AIB, followed by 20 μM AlCl_3_ for 30min. (D) Thirty root apexes were pretreated with 10 μM ACC and 50 μM malate for 6h, and exposed to 20 μM AlCl_3_ for 30min. The root tips were washed for 30min in control solution after Al treatments and then the Al concentrations were determined by ICP-OES. Data are the mean ± SE of four replicates and bars with different letters indicate significant difference at *P*<0.05 tested with SAS Software.

Malate released from root apices acts as a ligand to complex external Al^3+^, thus minimizing the toxic effect of Al^3+^ on root growth by preventing accumulation of Al^3+^ in root apices (Delhaize *et al.*, 1993*b*). The inhibition of malate efflux by ethylene should lead to greater accumulation of Al in the root apices. To test this hypothesis, Al content in root apices was measured by staining roots with the Al-sensitive probe haematoxylin, as well as quantitatively determined Al contents in root apices by inductively coupled plasma optical emission spectrometry (ICP-OES). Figure 6 shows that Al was mainly accumulated in the quiescent zones in the absence of ACC, and that exposure of ET8 seedlings to ACC led to an enhanced accumulation of Al in these areas as well as in the differentiation zone. A similar increase in Al content in ET8 root apices after treatment with ACC was observed (Fig. 6A). Moreover, the increase in Al content in root apices by ACC pretreatment increased with increasing pretreatment time (Fig. 6B). For example, Al content in the root apices was increased by 32%, 95%, and 216% after exposure to ACC for 2, 4, and 6h before application of Al, respectively, whereas Al content in root apices exposed to solution without ACC showed relatively lower Al content (Fig. 6B). In contrast to treatment with ACC, Al contents in root apices were significantly reduced by AVG and AIB (Fig. 6C). The involvement of ethylene in Al accumulation in root apices was further evaluated by comparing the effect of exogenous application of malate on Al content in root apices with that of ACC. Exogenous application of malate significantly reduced Al content in root apices, whereas ACC increased the Al content (Fig. 6D). The increase in Al content by ACC was markedly suppressed by malate (Fig. 6D).

### Ethylene inhibited root elongation similar to Al^3+^

**Fig. 7.**
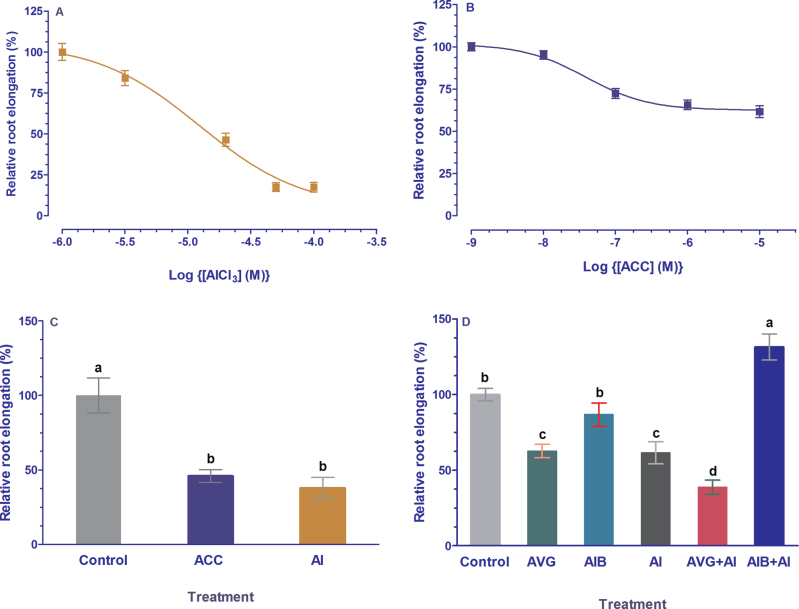
Effect of aluminium (Al), ethylene biosynthesis precursor (ACC) and ethylene synthesis inhibitors (AVG, AIB) on root elongation of ET8. (A) Root elongation in response to various concentrations of AlCl_3_. Roots of 5-day-old seedlings were incubated in solutions containing different concentrations of AlCl_3_ (0, 10, 20, 50, 100 μM) and elongation was measured by ruler after 24h. (B) Effect of varying concentrations of ACC on root elongation. Root elongation was determined after roots were treated with different concentrations of ACC (0, 0.01, 0.1, 1, 10 μM) for 24h. (C) Short-term effect of ACC and Al on root elongation. Five-day-old seedlings were fixed in 10-cm Petri dishes and roots were incubated for 1h in control solution (0.2mM CaCl_2_, pH 4.5) or treatment solutions containing 10 μM ACC or 20 μM AlCl_3_. Root elongation was measured by microscope. (D). Root elongation in response to ethylene synthesis inhibitors (AVG, AIB). Roots of five-day-old seedlings were pretreated with control solution or the solutions containing 10 μM AVG or 50 μM AIB for 6h, and then exposed to 0 μM or 20 μM AlCl_3_ for another 2h. Root elongation were measured after exposing to AlCl_3_ for 2h by microscope. Values are given as means ± SE of at least eight independent measurements. The different letters indicate significant difference at *P*<0.05 tested with SAS.

The most distinct symptom of Al^3+^ toxicity is inhibition of root elongation. Efflux of organic anions alleviates the Al^3+^-induced inhibition of root growth by complexing toxic Al^3+^ in the rhizosphere. Ethylene gas and ethylene synthesis precursor ACC suppressed Al^3+^-induced malate efflux from ET8 root apices and enhanced Al accumulation in root tips (Figs 2 and 6), suggesting that ethylene may be involved in the Al^3+^-induced inhibition of root elongation. To test this hypothesis, we compared the effect of ACC and AlCl_3_ on root elongation. As shown in Fig. 7, treatment with ACC and AlCl_3_ for 24h markedly suppressed root elongation. The IC_50_ values for inhibition of root elongation by Al^3+^ and ACC were 12.4 μM and 0.04 μM, respectively (Fig. 7A, B), suggesting that root elongation is more sensitive to ACC than Al^3+^. A similar rapid inhibition of root elongation by ACC also occurred. For instance, root elongation was inhibited by 54% and 62% after exposure to 10 μM ACC and 20 μM ACC (pH 4.5), respectively, for 1h (Fig. 7C). Root elongation was inhibited by AVG and Al^3+^ when treated alone, whereas AIB had no effect on root elongation in the absence of Al^3+^ (Fig. 7D). However, pretreatment of wheat roots with AIB reversed Al^3+^-induced inhibition of root elongation, leading to greater root elongation than control roots that were exposed to control solution (Fig. 7D). In contrast, pretreatment with AVG potentiated Al^3+^-induced suppression of root elongation (Fig. 7D).

### Ethylene up-regulated *TaALMT1* expression

**Fig. 8.**
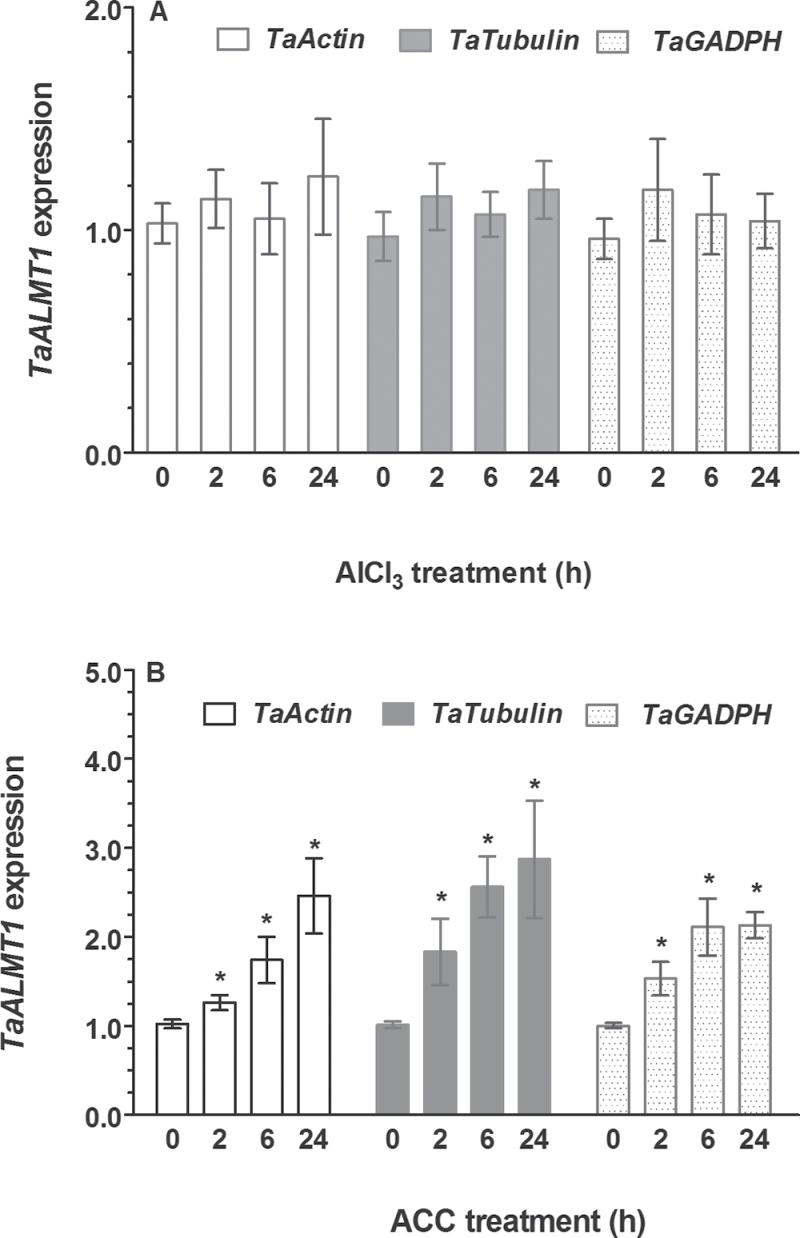
Effect of Al (A) and ethylene biosynthesis precursor (ACC) (B) on *TaALMT1* expression of ET8 root apices. Expression of *TaALMT1* was determined after exposure of root apices to 20 μM AlCl_3_ and 10 μM ACC for varying time periods (0, 2, 6, 24h). The relative mRNA level was normalized to the mRNA in roots grown in control solution. Three reference genes, *TaActin*, *TaTubulin*, and *TaGADPH*, were used in determination of effect of ACC and Al on *TaALMT1* expression. Data are the means ± SE of three replicates and an asterisk indicates significant difference with control at *P*<0.05.

Previous studies demonstrated that *TaALMT1* was expressed constitutively in wheat roots (Sasaki *et al.*, 2004). To test whether the suppression of Al-induced malate efflux by ethylene is related to TaALMT1 at the transcriptional level, the effect of ACC and AlCl_3_ on *TaALMT1* expression was investigated. As shown in Fig. 8, regardless of the reference genes used in qRT-PCR, the expression of *TaALMT1* was enhanced after exposure to ACC, whereas expression of *TaALMT1* in ET8 root apices was not responsive to Al^3+^ (Fig. 8). These results suggest that regulation of *ALMT1*-mediated malate efflux from wheat root apices is unlikely to occur at the transcriptional level.

## Discussion

There have been numerous studies reporting the involvement of ethylene in morphological responses of plants to nutrient deficiency and metal toxicity (Jung *et al.*, 2009; Tian *et al.*, 2009; Sun *et al.*, 2007, 2010). In addition to modulation of root morphology, emerging evidence indicates ethylene may also play a regulatory role in physiological processes in response to mineral stress (Nagarajan and Smith, 2012; Iqbal *et al.*, 2013). Our previous studies showed that Al^3+^ evoked a rapid and marked ethylene evolution in *L. japonicus* (Sun *et al.*, 2007) and *A. thaliana* (Sun *et al.*, 2010). In the present study, we found that Al^3+^ also evoked an evolution of ethylene from root apices of an Al-tolerant ET8 wheat genotype (Fig. 1). We evaluated the role of Al-induced ethylene production in the overall tolerance of ET8 to Al by experimentally manipulating the endogenous ethylene level using ethylene gas, ethylene donors, ethylene biosynthesis precursor, and ethylene synthesis inhibitors. Our results reveal that ethylene negatively regulates Al^3+^-induced malate efflux from root apices of Al-tolerant wheat plants and from tobacco BY2 cells expressing *TaALMT1* (Figs 2 and 5). We further demonstrate that ethylene may act on the TaALMT1 protein as shown by a similar, non-additive effect of ethylene and anion channel blocker niflumic acid on malate efflux from wheat root apices (Fig. 3). These findings, together with the observations that treatment with ethylene synthesis precursor ACC and ethylene synthesis inhibitors (AVG, AIB) enhanced and reduced accumulation of Al in the root apex, respectively (Fig. 6), provide evidence in support of the involvement of ethylene in Al tolerance in wheat by regulating ALMT1-mediated malate efflux. The enhanced accumulation of Al in root apices treated with ACC owing to suppression of malate efflux can also account for the results that inhibition of ethylene production by antagonists of ethylene synthesis (AIB) alleviated Al-induced arrest of root elongation. Antagonist of ethylene biosynthesis AVG enhanced Al-induced malate efflux and reduced Al accumulation in root apices (Figs 2 and 6), but Al-induced suppression of root elongation was potentiated, rather than alleviated, by treatment with AVG (Fig. 7D). This observation suggests that AVG may have other effects on root elongation in addition to the inhibition of ethylene biosynthesis. A recent study shows that AVG can inhibit root growth by affecting nitrogen metabolism (Lemaire *et al.*, 2013). A similar explanation may also account for our observation in the present study. Therefore, results obtained from effects of AVG on plant growth cannot be conclusively attributed to an ethylene effect.

In a recent study, Yang *et al.* (2011) reported that Al^3+^-induced malate efflux from root apices of ET8 wheat plants was stimulated by exogenous application of IAA, and that endogenous IAA content was enhanced owing to inhibition of IAA oxidase activity by Al^3+^. Similar to our results, the authors found that the Al^3+^-induced malate efflux is inhibited by antagonists of auxin polar transport (TIBA; 2,3,5-triiodobenzoic acid, and NPA; naphthylphthalamic acid), and anion channel blockers (niflumate and A-9-C) (Yang *et al.*, 2011). A close crosstalk between ethylene and auxin in regulation of root growth and development has been reported in the literature (see review of Stepanova and Alonso, 2009). Our previous results showed that ethylene evoked by Al^3+^ via up-regulating ACS and ACO at the transcriptional level may act as an up-stream signal to alter auxin transport and distribution in roots, leading to the arrest of root elongation (Sun *et al.*, 2010). Whether a similar interaction between ethylene and auxin in regulation of Al^3+^-induced malate efflux operates warrants further investigation by experimentally manipulating ethylene and/or auxin production and distribution with antagonists of ethylene synthesis and perception, auxin polar transport and exogenous application of auxin and ethylene.

The inhibitory effect of ethylene on Al^3+^-induced malate efflux is unlikely to occur at the transcriptional level as ACC did not suppress expression of *TaALMT1*, rather an up-regulation of *TaALMT1* in response to ACC was observed (Fig. 8). Anion channel antagonist NA that blocks Al^3+^-activated ALMT1 channels (Zhang *et al.*, 2008) and Al^3+^-induced malate efflux (Ryan *et al.*, 1995) exhibited similar IC_50_ value to ACC in their effect on malate efflux (Fig. 3). Moreover, we found that ethylene and NA had non-additive effect on Al^3+^-induced malate efflux (Fig. 3). These results suggest that ethylene may act as a channel blocker to depress TaALMT1-mediated malate efflux. Alternatively, ethylene may regulate ALMT1 by preventing its activation by Al^3+^. Our observation that ethylene inhibited Al^3+^-induced malate efflux from tobacco BY2 cells expressing TaALMT1 seems to be in line with these hypotheses. However, to elucidate the mechanisms responsible for suppression of Al^3+^-induced malate efflux, additional experiments will be needed such as probing the interaction between Al^3+^ and ethylene on ALMT1-mediated currents with electrophysiological techniques.

There are many reports showing that ethylene can regulate expression of genes encoding membrane transporters such as phosphate, nitrate, and iron (Li *et al.*, 2011; Tian *et al.*, 2009; Garcia *et al.*, 2010), but few studies have focused on the effect of ethylene on transport of ions at protein and cellular levels. Leblanc *et al.* (2008) showed that treatment of oilseed rape seedlings with ACC and AVG reduced and enhanced nitrate uptake, respectively, by monitoring ^15^N uptake. They suggested that a posttranscriptional regulation of nitrate transporters may be involved in the regulation of nitrate transport by ethylene (Leblanc *et al.*, 2008). Our previous study showed that ethylene can increase the concentrations of cytosolic Ca^2+^ by activating Ca^2+^-permeable cation channel in tobacco cells (Zhao *et al.*, 2007). Although there has been no report showing the involvement of cytosolic Ca^2+^ activity in regulation of Al^3+^-activated ALMT1-mediated malate efflux in the literature so far, we cannot rule out the possibility that inhibition of ALMT1-mediated malate efflux by ethylene may occur through changes in cytosolic Ca^2+^ activity.

Recent studies shed some lights on the mechanisms by which Al^3+^ activates anion channels. For instance, in *Arabidopsis*, upstream transcription factors *AtSTOP1* and *AtWRKY64* were reported to be involved in Al^3+^-induced expression of *AtALMT1* (Iuchi *et al.*, 2007; Sawaki *et al.*, 2009; Ding *et al.*, 2013). In wheat, Al^3+^-induced malate efflux is mainly controlled by ALMT1 protein, the expression of which is constitutive and not induced by Al (Sasaki *et al.*, 2004). A post-transcriptional regulation of TaALMT1 by Al^3+^ seems to be an important mechanism (Furuichi *et al.*, 2010; Ryan and Delhaize, 2010). Although the extracellular C-terminal domain is proposed to be a key site interacting directly with external Al^3+^ and the structural integrity of TaALMT1 is considered to be involved in Al^3+^-sensing of TaALMT1, the mechanism underlying the activation of ALMT1 by Al^3+^ remains unknown (Furuichi *et al.*, 2010; Ligaba *et al.*, 2013). It is unclear whether Al^3+^ activates the ALMT1 channel directly or through signalling molecules. There is emerging evidence suggesting that reversible phosphorylation may also be involved in Al^3+^-induced malate efflux from wheat roots (Osawa and Matsumoto, 2001) and *Arabidopsis* roots (Kobayashi *et al.*, 2007) as shown by the inhibition of Al-activated malate efflux by protein kinase inhibitors (K252a and staurosporine). Ligaba *et al.* (2009) demonstrated that malate current in *Xenopus laevis* oocytes expressing *TaALMT1* is regulated by protein kinase C-mediated phosphorylation. Moreover, Al can induce a 48kDa protein kinase in wheat roots and coffea (*Coffea arabica*) suspension cells (Osawa and Matsumoto, 2001; Martinez-Estevez *et al.*, 2001). It is conceivable that ethylene may regulate phosphorylation of TaALMT1 by targeting a protein kinase, because protein kinases and phosphorylation play important roles in ethylene signalling cascades (Ju *et al.* 2012). For example, five ethylene receptors identified in *Arabidopsis* possess kinase activity (Gamble *et al.*, 1998). The receptors interact with a Raf-like protein kinase CTR1, a negative regulator of the ethylene signalling pathway (Kieber *et al.*, 1993), leading to inactivation of downstream signalling components EIN2 and EIN3 (Alonso *et al.*, 1999). Ethylene binding results in the inactivation of the receptor–CTR1 complex and the accumulation of EIN3 and EIN3-like transcription factors EILs in the nucleus (Guo and Ecker, 2003), which in turn activate and repress hundreds of genes by initiating a transcriptional cascade (Alonso *et al.*, 1999). The negative regulation of Al-dependent malate efflux by ethylene suggests that some components of ethylene signalling cascades may interact with ALMT1 directly or indirectly. Alternatively, ethylene elicited by Al^3+^ may target ALMT1 by interacting with other unknown signalling molecules, leading to the observed suppression of Al-induced malate efflux. Future work using mutants of ethylene biosynthesis and signalling and *Xenopus* oocytes expressing *TaALMT1* may unravel the molecular mechanism underlying the interaction between Al and ethylene in modulation of malate efflux.

In summary, we show that pretreatment of wheat roots with ethylene gas and ACC suppressed Al^3+^-induced malate efflux. The suppression of Al-induced malate efflux by ethylene is likely to result from inhibition of ALMT1-mediated malate efflux as shown by a similar effect of ethylene on Al-induced malate efflux from tobacco cells expressing *TaALMT1*. The suppression of ALMT1-mediated malate efflux by ethylene may occur through post-transcriptional regulation of ALMT1 because ethylene enhanced rather than inhibited expression of *TaALMT1*. Although the mechanism by which ethylene inhibits Al-dependent malate efflux remains to be elucidated, our findings demonstrate that ethylene may be an important component in the regulation of ALMT1-dependent malate efflux. Finally, our results show that the effect of ethylene on ALMT1-dependent malate efflux occurs at the post-transcriptional level. Therefore, future research to decipher the molecular mechanisms underlying the regulation of ALMT1-dependent malate efflux by Al and ethylene at protein level is warranted.
